# Prevalence and Antibiotic Resistance Pattern of *Salmonella* Isolated from Caecal Contents of Exotic Chicken in Debre Zeit and Modjo, Ethiopia

**DOI:** 10.1155/2020/1910630

**Published:** 2020-01-18

**Authors:** Destaw Asfaw Ali, Belege Tadesse, Aragaw Ebabu

**Affiliations:** ^1^College of Veterinary Medicine and Animal Science, University of Gondar, Gondar, Ethiopia; ^2^School of Veterinary Medicine, Wollo University, Dessie, Ethiopia; ^3^College of Veterinary Medicine and Agriculture, Addis Ababa University, Addis Ababa, Ethiopia

## Abstract

A cross-sectional study was conducted between December, 2013, and May, 2014, to determine the prevalence and antibiotic resistance feature of *Salmonella* isolated from broilers slaughtered in Debre Zeit and Modjo towns, Ethiopia. A total of 384 caecal content samples were collected for microbiological examination following the standard techniques and procedures outlined by the International Organization for Standardization to isolate *Salmonella*. The sensitivity of the isolates subjected to nine antimicrobials was tested by the Kirby–Bauer disk diffusion method. The overall prevalence of *Salmonella* was 14.6%, and its occurrence differ significantly by farm (*p* < 0.05). The occurrence of the bacteria was not statistically different in the midland (15.2%) and lowland (13.3%) (*p* > 0.05) and between males (13.5%) and females (15.6) (*p* > 0.05). Of the 50 isolates, 48 were resistant to at least one drug. Multidrug resistance was recorded in 43 (86.0%) of the isolates. The study demonstrated considerable prevalence and high antimicrobial resistant *Salmonella* in exotic chicken and indicates the potential importance of chickens as source of foodborne salmonellosis and multiple antimicrobial resistance of *Salmonella*. Improving the hygienic practice of farms could help to reduce the occurrence of *Salmonella* in farms. Further studies are needed to describe the risk factors associated with the emergence of drug-resistant *Salmonella* in chicken.

## 1. Introduction

Despite global improvements in public health facilities, bacterial infections still remain an important public health problem worldwide. Salmonellosis is one of the main foodborne zoonotic and animal husbandry problem throughout the world [1, 2]. The bacteria cause foodborne poisoning in humans, mainly through animal products that include poultry, cattle, and pig products [3, 4].

*Salmonella* is facultative anaerobic Gram-negative rod that grows optimally at 35°C to 37°C and catabolizes a variety of carbohydrates into acid and gas in addition to H_2_S gas production [5]. The genus comprises two species: *Salmonella enterica* and *Salmonella bongori* [6].

Salmonellosis has been recognized in all countries but seems to be most prevalent in areas of intensive animal husbandry, especially poultry and swine production. Although primarily an intestinal bacteria, *Salmonella* is widespread in the environment and commonly found in farm effluents, human sewage, and in any material subject to fecal contamination [7]. The natural habitat of *Salmonella* may be divided into three categories: (i) highly adapted to men and agents of typhoid fever; (ii) highly adapted to animals responsible for animal paratyphoid; and (iii) most of the serovars that affect men and animals [8].

In developing countries, domestic chickens live in close contact with humans in both urban and village communities, often being housed overnight in the family home [9]. Infection of the chicken leads to fecal shedding into the environment. Contamination of meat and eggs is the main chain of human *Salmonella* infections in addition to the contact of workers in poultry farms and slaughtering houses [10]. The risk development of antibiotic resistance in human and animal is one of the main reasons for control in animals [11].

Antimicrobial resistance is a global public health problem [12, 13]. Resistance to antimicrobials could be due to three basic mechanisms: (i) modification of the antibiotic by decreasing absorption or increasing efflux of the antibiotic by using their enzymes; (ii) change in the target site of the antibiotic; and (iii) acquisition of the ability to break or modify the antibiotic [14]. Several lines of evidence demonstrate that the use of antimicrobial agents in food animals contributes to the emergence and dissemination of antimicrobial resistance in food borne *Salmonella* [15]. In recent years, antimicrobial resistance is a serious problem in the treatment of *Salmonella* infection [16, 17].

*Salmonella* infections of food animals play an important role in public health and particularly in food safety, as food products of animal origin are considered to be the major source of human *Salmonella* infections [18]. Cross-contamination during food processing is also monitored as contamination by healthy food handlers. In recent years, it is reported that livestock and their products can contribute to as much as 96% [19] of the total *Salmonella* infection in humans [20].

In Ethiopia, several factors including under and malnutrition, HIV-AIDS, the unhygienic living circumstances, and the close relations between humans and animals may substantially contribute to the occurrence of salmonellosis. Although surveillance and monitoring systems are not in place and its epidemiology is not described, qualitative and quantitative syntheses of previous studies could shed light on the occurrence of the disease and the major serotypes that frequently cause infections. In a recent study on diarrheal patients, the prevalence of the disease was estimated at 8.22% [21], and in patients with febrile illnesses, typhoid fever was recorded in 5.85% of the patients with a higher occurrence in children aged 3 to 14 years (6.6%) compared with children aged 15 to 17 years (1.1%) [22]. These reports imply the need for a policy to promote public hygiene and regularly screen individuals in contact with food items for public consumption.

Despite salmonellosis imposes a significant cost to societies, in many countries, few countries report the data on economic cost of the disease [11, 23]. The disease offers one of the greatest scopes for research particularly when relating to developing countries [24]. Although studies conducted in Ethiopia indicated the considerable importance of *Salmonella* in animals and humans [25, 26], studies on chicken are limited. Therefore, this study was undertaken with the objective to determine the prevalence and antibiotic resistance features of *Salmonella* isolated from the caecal contents of broilers.

## 2. Materials and Methods

### 2.1. Study Areas

The study was conducted in Debre Zeit and at Modjo, Ethiopia. Debre Zeit and Modjo are found in Oromia regional state 47 and 72 km South East of Addis Ababa, respectively. Debre Zeit is located at 9°N and 39°E, at an altitude of about 1900 m above sea level. Modjo is located at 8.3°N and 39°E, at an altitude of 1774 m above sea level. The areas experience a bimodal rainfall with the main rainy season extending from June to September. Debre Zeit and Modjo have annual average rain fall of 800 mm and 776 mm, average temperature of 18.7°C and 19.4°C, and mean relative humidity of 61.3% and 59.9%, respectively [27].

### 2.2. Study Design

The study was cross-sectional and conducted from December 2013 to May 2014. The animals were apparently healthy chicken slaughtered at commercial processing plants and private producers. The chickens were rose breed maintained under intensive management system and are of the same age (45–50 days) groups. The sample size was determined according to Thrusfield [28] with an expected prevalence of 50%, 95% confidence interval, and 5% absolute precision. Accordingly, a total of 384 chickens were sampled: 133 from Alema Farms commercial processing plant, 120 from Modjo poultry slaughtering house, and 131 from three other private holders in Debre Zeit. Sampling was systematic and random. The caecum of each chicken was punctured with sterile scalpel, and about 5 grams of caecal contents were collected in labeled universal bottles. Samples were transported to the Microbiology laboratory of the Collage of Veterinary Medicine and Agriculture, Addis Ababa University, by using an ice box. The sample was refrigerated at 4°C and processed within 24 hours.

### 2.3. Isolation and Identification

The isolation and identification of *Salmonella* was according to the techniques recommended by the International Organizations for Standardization (ISO-6579) [29] with Global *Salmonella* Survey (GSS) and WHO guidelines [30]. All the media used were prepared according to the instructions of the manufacturer.

#### 2.3.1. Culture Methods

*(1) Nonselective Preenrichment*. The refrigerated samples were thawed at room temperature, and 5 grams of the caecal contents were preenriched with 45 ml of buffered peptone water at a ratio of 1 : 9 BPW (HiMedia M1494, Mubi, India). The suspension was harmonized by mixing using a vortex and incubated for 24 hr at 37°C.

*(2) Selective Enrichment*. A portion (1 ml) of the preenriched cultured was transferred to 10 ml of selenite-F (Titan TM389, Biotech, India) broth, and another 0.1 ml portion was transferred to 10 ml of RV broth (HiMedia M880, Mubi, India) and incubated at 37°C and 42°C for 24 hours, respectively.

*(3) Plating Out and Identification*. From each selective enrichment broth media (RV and SF), a loop full of the suspension was inoculated on to xylose lysine deoxycholate (XLD) (HiMedia M031, Mubi, India) and Brilliant Green (BG) (Titan TM951, Biotech, India) agars and incubated at 37°C for 24 hr. The incubation was prolonged to 48 hr for those who did not show any growth during the 24 hr incubation. Typical colonies, having a slightly transparent zone of reddish color and a black center (XLD), and atypical lactose-positive salmonellae, yellow with or without blackening, from XLD and BG plate agars were isolated and subcultured on nutrient agar (Oxoid CM0003, Basingstoke, England).

#### 2.3.2. Biochemical Tests

Biochemical tests were performed by using the following tests: triple sugar iron agar (TSI) (Oxoid CM0277, Basingstoke, England), lysine iron agar (LIA) (Oxoid CM381, Basingstoke, England), urea broth (Oxoid CM53, Basingstoke, England), Voges Proskauer (VP) (Himedia M070I, Mumbai, India), tryptone broth (Himedia M463, Mumbai, India) for indole test.

#### 2.3.3. Antimicrobial Tests

The susceptibilities of the isolates to antimicrobials were tested by using the Kirby–Bauer disk diffusion method. The isolates were tested for the following antibiotics: ampicillin (AMP) (10 *μ*g/ml), streptomycin (STR) (10 *μ*g/ml), kanamycin (KAN) (30 *μ*g/ml), ceftriaxone (CRO) (30 *μ*g/ml), chloramphenicol (CHL) (30 *μ*g/ml), oxytetracycline (OXT) (30 *μ*g/ml), sulfamethoxazole-trimethoprim (SXT) (25 *μ*g/ml), nalidixic acid (NAL) (30 *μ*g/ml), and amoxicillin (AMC) (30 *μ*g/ml), all from Oxoid, England. The isolates were inoculated to brain heart infusion broth (Oxoid CM0225, Basingstoke, England) and incubated for 8 hours at 37°C till the turbidity of the suspension equals to that of a 0.5 McFarland standard. Plates of Mueller Hinton agar (Oxoid CM0337 Basingstoke, England) were seeded with socked inoculum of sterilized cotton swabs. Antimicrobial discs were placed on the agar surface at about 2 cm apart. The plates were incubated at 37°C for 24 hr, and the diameter of the zones of inhibition was measured by using a caliper. The measurements were compared with zone size interpretative chart furnished by Clinical Laboratory and Standard Institute document M100-S23 (M02-A11) [31], and zones were graded as susceptible, intermediate resistance, and resistant.

### 2.4. Statistical Analysis

The data were entered into a Microsoft Excel 2007 and imported to SPSS version 20 for analysis. Descriptive statistics was used to describe the data. Pearson's chi-square (*χ*^2^) test was used to test associations. Alpha was set at 0.05.

## 3. Results

### 3.1. Prevalence of *Salmonella*

*Salmonella* was recovered in 56 (14.6%) of the samples. The bacteria were isolated in 28 (21.1%), 16 (12.2%), and 12 (10.0%) of the samples that originated from Alema, Aleka Amba, and Modjo farms, respectively (Table 1). The occurrence of the bacteria differed significantly by farm (*p* < 0.05). Its occurrence was higher at Alema farms but not significantly different between Aleka Amba and Modjo farms (*χ*^2^ = 0.31; *p*=0.578). The occurrence of *Salmonella* did not differ by altitude (*p* > 0.05) and sex (*p* > 0.05) (Table 2).

### 3.2. Antibiotic Resistance

#### 3.2.1. Mono Drug Resistance

The antimicrobial susceptibility features of the isolates are given in Table 3. The highest resistance was recorded for oxytetracycline (82.0%) followed by ampicillin (70.0%). Resistance to kanamycin and ceftriaxone were observed in 26.0% and 6.0% of the isolates, respectively. Intermediate resistance to each of the tested drugs was recorded in four or more of the tested isolates. Only two isolates were sensitive to all drugs, and five were resistant to a single drug.

#### 3.2.2. Multidrug Resistance

The multidrug resistance features of the isolates are shown in Table 4. Of the tested isolates, 43 (86.0%) were resistant to two or more (up to seven) antimicrobials and 26 multidrug resistance profiles were observed. The number of isolates resistant to five drugs was higher followed by two drug-resistant isolates. The OXT, AMP, CHL, NAL, SXT, and STR (4/43) phenotype occurred more frequently followed by the OXT, AMP, CHL, NAL, SXT, and KAN (3/43) phenotype.

## 4. Discussion

The overall prevalence of *Salmonella* was 14.6% and differed by location. The prevalence difference in these sites could be due to differences in environmental contamination or management systems. However, the prevalence difference between Aleka Amba and Modjo farms did not differ statistically (*p* > 0.05). The prevalence difference of isolates between midland and lowland was 15.2% and 13.3%, respectively, while that of sex was 15.6% in female and 13.5% in male who did not also differ statistically (*p* > 0.05), and hence, there was no association between these risk factors and the prevalence of the pathogen.

The overall prevalence of *Salmonella* was 14.6%. The result is in agreement with the 16.1% prevalence estimate from faecal and cloacal swaps of in intensive poultry farms in Hawassa [32]. Comparable results were also reported in Egypt [33] and in Nepal [34]. By contrast, a higher prevalence in Jima (41.9%) was recorded [35], and a lower prevalence (9.3%) in East Showa, Ethiopia [26], was recorded. The differences in the prevalence estimates could be due to differences in environmental contamination, management systems, breed, sample size, and method of isolation.

Except two isolates, all were resistant to at least a single antimicrobial. A considerably higher proportion of the isolates 43 (86.0%) was resistant to two or more of the antimicrobials, and 26 multidrug resistance profiles were observed. Multidrug resistant is considered when it is simultaneously resistant to two or more drugs [36]. The number of isolates resistant to five drugs was higher followed by two drug-resistant isolates. Most of the antimicrobial drugs for which *Salmonella* isolates were resistant in this study were reported earlier from Ethiopia from different food animals, food products, and humans [37–40]. The types of antibiotic resistant for the isolates in this study are also in agreement with different reports in other parts of African [41] and European countries [42, 43].

In developing countries, antimicrobial resistance levels have markedly increased, and this could be due to the indiscriminate and widespread uses of antimicrobials both in the veterinary and public health practices as access to antimicrobials is easy and can be purchased without prescription [44–46]. *Salmonella* isolates resistant to the relatively cheaper and commonly available antimicrobials is disturbing as the resistance causes more expensive therapies and longer duration of sickness. The resistant pattern of *Salmonella* in poultry confers to drugs such as ceftriaxone that used for treating salmonellosis in humans. This suggests that chicken could be a source for multidrug-resistant salmonellosis in humans [47].

The present work contributes considerably towards understanding of the prevalence of *Salmonella* and the antibiotic resistance features of chicken isolates in Ethiopia. Serotyping of the isolates will complement this report and enable us understanding the serotypes.

## 5. Conclusion and Recommendations

The overall prevalence of *Salmonella* in chicken is considerable, and the antibiotic resistance feature of the subjected isolates is recorded high. Chicken are potential sources of foodborne salmonellosis and antimicrobial resistant *Salmonella* that pose a high risk to both animals and humans. Awareness should be created to avoid indiscriminate use of antimicrobials. There should be intense and effective communication between veterinary organizations and human health care providers to control spread of salmonellosis and to target antibiotic resistance. Further studies are needed to describe the occurrence of *Salmonella* in chicken and the risk factors that favor the emergence of drug-resistant isolates.

## Figures and Tables

**Table 1:** Overall prevalence of *Salmonella* by sites of samples examined.

Farms	Number examined	Number positive (%)	*χ* ^*2*^	df	*p* value
Alema	133	28 (21.1)	7.083	2	0.029
Aleka Amba	131	16 (12.2)			
Modjo	120	12 (10.0)			
Total	384	56 (14.6)			

df = degree of freedom.

**Table 2:** Prevalence of *Salmonella* in slaughtered chicken by risk factors.

Risk factors		Number of samples	Prevalence (%)	*χ* ^*2*^	df	*p* value
Altitude	1900 masl	264	44 (15.2)	2.94	1	0.086
	1774 masl	120	12 (13.3)			

Sex	Female	192	30 (15.6)	0.334	1	0.563
	Male	192	26 (13.5)			

masl = meter above sea level; df = degree of freedom.

**Table 3:** Number (%) of *Salmonella* isolates resistant to antimicrobials.

Antibiotics	Susceptible (%)	Intermediate (%)	Resistant (%)
AMC	25 (50.0)	13 (26.0)	12 (24.0)
AMP	11 (22.0)	4 (8.0)	35 (70.0)
CHL	25 (50)	8 (16.0)	17 (34.0)
CRO	39 (78.0)	8 (16.0)	3 (6.0)
KAN	13 (26.0)	24 (48.0)	13 (26.0)
NAL	14 (28.0)	7 (14.0)	29 (58.0)
OXT	4 (8.0)	5 (10.0)	41 (82.0)
STR	19 (36.0)	16 (32.0)	15 (30.0)
SXT	16 (32.0)	6 (12.0)	28 (56.0)
Total	166 (36.9)	91 (20.2)	193 (42.9)

**Table 4:** Multiple antimicrobial resistance profiles of *Salmonella* isolates (*n* = 43).

Number of antimicrobials	Resistance pattern (no of isolates)	Number of isolates (%)
Two	AMP, OXT (3); AMP, AMC (1); AMC, KAN (1)	9 (18)
	OXT, NAL (2); CHL, NAL (1); SXT, NAL (1)	

Three	AMP, AMC, OXT (4); AMC, OXT, SXT (3)	7 (14)

Four	AMP, AMC, OXT, KAN (1); AMP, AMC, OXT, SXT (1);	8 (16)
	SXT, OXT, AMP, STR (1); SXT, OXT, AMP, NAL (1);	
	SXT, OXT, NAL, STR (1); OXT, CHL, NAL, AMP (2),	
	OXT, CHL, NAL, STR (1)	

Five	AMP, AMC, OXT, SXT, NAL (1); AMP, AMC, OXT, CRO, STR (1)	11 (22)
	AMP, OXT, CHL, SXT, NAL (2); AMP, OXT, SXT, NAL, KAN (2)	
	AMP, OXT, CHL, NAL, KAN (1); AMP, OXT, CHL, SXT, STR (1)	
	AMP, OXT, NAL, SXT, STR (2); OXT, CHL, SXT, NAL, KAN (1)	

Six	OXT, AMP, CHL, NAL, SXT, STR (4);	7 (14)
	OXT, AMP, CHL, NAL, SXT, KAN (3)	

Seven	OXT, AMP, CHL, NAL, SXT, KAN, STR (1)	1 (2)

## Data Availability

The data used to support the findings of this study are included within the article in the frequency table.
